# Open-source analytical pipeline for robust data analysis, visualizations and sharing in crop breeding

**DOI:** 10.1186/s13007-022-00845-7

**Published:** 2022-02-05

**Authors:** Waseem Hussain, Mahender Anumalla, Margaret Catolos, Apurva Khanna, Ma. Teresa Sta. Cruz, Joie Ramos, Sankalp Bhosale

**Affiliations:** Rice Breeding Innovation Platform, International Rice Research Institute (IRRI), Los Banos, Laguna Philippines

**Keywords:** Rice, Breeding analytics, Open-resource, Interactive visualizations, Reproducibility

## Abstract

**Background:**

Developing a systematic phenotypic data analysis pipeline, creating enhanced visualizations, and interpreting the results is crucial to extract meaningful insights from data in making better breeding decisions. Here, we provide an overview of how the Rainfed Rice Breeding (RRB) program at IRRI has leveraged R computational power with open-source resource tools like R Markdown, *plotly*, LaTeX, and HTML to develop an open-source and end-to-end data analysis workflow and pipeline, and re-designed it to a reproducible document for better interpretations, visualizations and easy sharing with collaborators.

**Results:**

We reported the state-of-the-art implementation of the phenotypic data analysis pipeline and workflow embedded into a well-descriptive document. The developed analytical pipeline is open-source, demonstrating how to analyze the phenotypic data in crop breeding programs with step-by-step instructions. The analysis pipeline shows how to pre-process and check the quality of phenotypic data, perform robust data analysis using modern statistical tools and approaches, and convert it into a reproducible document. Explanatory text with R codes, outputs either in text, tables, or graphics, and interpretation of results are integrated into the unified document. The analysis is highly reproducible and can be regenerated at any time. The analytical pipeline source codes and demo data are available at https://github.com/whussain2/Analysis-pipeline.

**Conclusion:**

The analysis workflow and document presented are not limited to IRRI’s RRB program but are applicable to any organization or institute with full-fledged breeding programs. We believe this is a great initiative to modernize the data analysis of IRRI’s RRB program. Further, this pipeline can be easily implemented by plant breeders or researchers, helping and guiding them in analyzing the breeding trials data in the best possible way.

## Background

The International Rice Research Institute (IRRI), established in the 1960s, is the world’s premier research organization dedicated to rice science. Rainfed rice breeding (RRB) at IRRI started since the establishment of the institute and is continuously committed to innovate and develop improved rice germplasm for improving the livelihood of farmers encountering challenging climates [1]. Currently, the ongoing rice breeding project, “Accelerated Genetic Gains in Rice Alliance” at IRRI, funded by the Bill and Melinda Gates Foundation (BMGF), is mandated to modernize breeding strategies and framework to increase the current rates of genetic gains in close collaboration with NARES network-partner’s across South Asia (India, Bangladesh, and Nepal), East and Southern Africa (Kenya, Mozambique, Tanzania, and Burundi).

Every year RRB at IRRI shares the breeding germplasm tolerant to drought, salt, heat, and submergence with the regional partner’s for phenotypic evaluation and, in return, receives raw phenotypic data from several trials at different locations. For instance, the RRB during the year 2019 received data from approximately 20 trials from the NARES partners. It is crucial to demystify data analysis for regional partner’s to make better breeding decisions and present the results in an easy and understandable format. Detailed documentation will contribute to a clear interpretation and understanding of results along with promoting collaborations. Furthermore, simultaneously analyzing and documenting the results has not been possible with readily available computational tools that require a ‘copy and paste’ system to document or report the highly error-prone results. Thus, we believe an immediate up-gradation of data analysis workflow is crucial to be more effective and enhance reproducibility [2]. The high-end improvement is necessary for conveniently documenting and sharing the result reports.

Technology advances have made data management, analysis, interpretation, visualization, and sharing more convenient. For example, R software [3] packages viz*., ggplot2* [4], *plotly* (https://plotly.com/), *DT* (https://rstudio.github.io/DT/) has made the data mining manageable and visualizations interactive and dynamic. Similarly, with *R Markdown* [5], data analysis can be turned into high-quality, reproducible reports in which codes, text, tables, graphics, and more are embedded in one unified document. Furthermore, the reports can be generated in various formats, including MS Word, PDF, HTML (Hyper-Text Markup Language), and more for seamless sharing (https://rmarkdown.rstudio.com/).

Here, we provide an overview of how the RRB program at IRRI has leveraged in R computational power with open-source resource tools of R Markdown, plotly, LaTeX [6] (https://www.latex-project.org/get/) and HTML to develop an analysis workflow of phenotypic data analysis, and re-designing it to a reproducible document for better interpretations, visualization and easy sharing with collaborators. The developed analysis workflow demonstrates how to pre-process and check data quality and perform robust data analysis using modern statistical tools and approaches. Besides developing this analytical pipeline and workflow, we showed how this workflow could be embedded into a well-descriptive document or report. In practice, we provide an open-source analytical pipeline with comprehensive details, procedures, and end-to-end steps. It integrates the analysis workflow, explanatory text with R codes, outputs either in text, tables, or graphics, and interpretation of results into a single document or, in simpler words, ‘everything is at one place. The complete and detailed description of results will act as a guide for phenotypic data analysis. It can be easily put into practice by the plant breeders and or plant researchers having a full-fledged breeding program.

## Overview of analysis workflow and pipeline

**Fig. 1:**
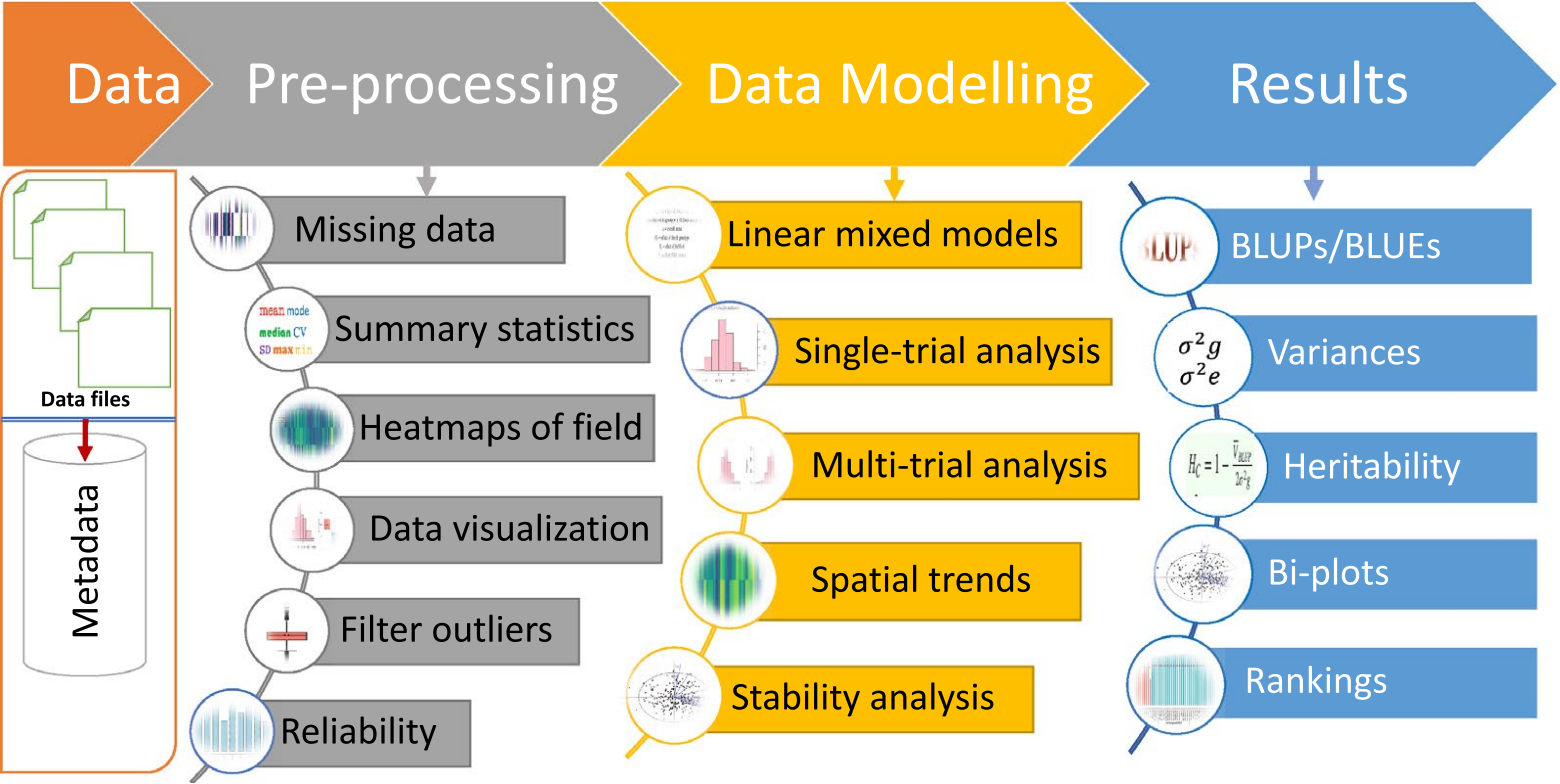
Schematic representation of data analysis workflow adapted in the current report. The four main steps involved in the analysis workflow process are **a** data importing, **b** data pre-processing, **c** data modeling, and **d** results generation. The main steps are divided into individual components required to develop a comprehensive and robust analysis pipeline

**Fig. 2:**
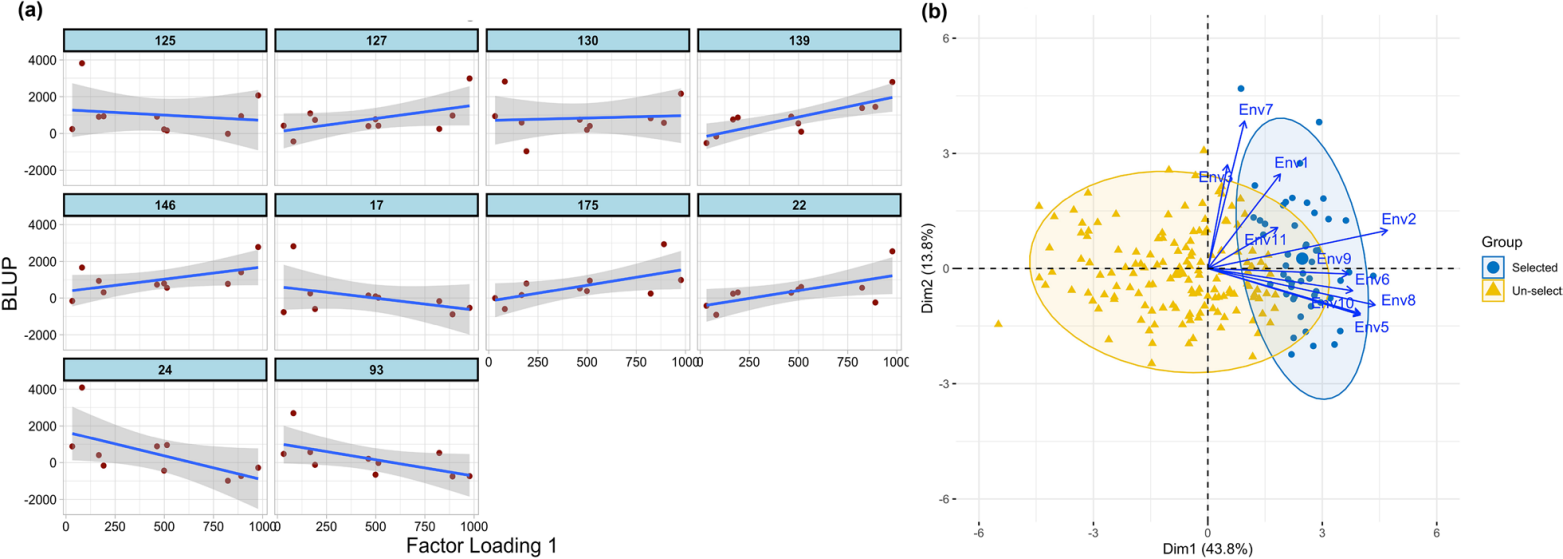
Results extracted from the MET analysis. **a** Latent regression plot for the top 10 genotypes using first factor estimated loadings. The solid blue line and the gray shade correspond to the latent regression line and the confidence interval of 95%, respectively. **b** Biplot of selected genotypes (in blue color) and un-selected genotypes (in yellow triangles) based on predicted breeding values adjusted across all environments based on factor analytic covariance structure. The blue lines with arrows show the environments and their correlations

Figure 1 illustrates the improved analysis workflow adopted in this study to analyze multi-environment trial data. The workflow is divided into four main components: data import, pre-processing and quality check, data analysis, and result extractions. In the pre-processing and quality check, we demonstrated a detailed procedure and instruction on checking the quality of data and ensuring only high-quality phenotypic data points are advanced for downstream analysis to get reliable estimates or predictions of genotypes. The sample document for this available is on GitHub (https://github.com/whussain2/Analysis-pipeline). For the data analysis step, we provide a detailed overview of how to analyze the data separately or jointly using linear mixed-model (LMM) approaches. The analytical pipeline is demonstrated both in the ASReml-R package and in the lme4 R package available on GitHub (https://github.com/whussain2/Analysis-pipeline). We applied mixed models ranging from basic to higher advanced models in separate-trial analysis accounting for experimental design factors and spatial trends. Similarly, in multi-environment trial (MET) data, we showed single-stage or two-stage analysis approaches ranging from basic models to higher advanced factor analytical models. In the results step, we demonstrated selecting the best model and using it to extract different results. Results including BLUPs, heritability, correlation and covariance matrix of environments, G x E BLUPs, principal component analysis (PCA) biplot-showing stability and relationship of environments, and latent regression plots to accesses the stability of genotypes (Fig. 2) were presented. All the instructions, R source codes, examples, and the data sets are freely available in the GitHub repository at https://github.com/whussain2/Analysis-pipeline. Additional resources on analyzing the MET data and checking the stability of genotypes are given in section 1.4 of the ASReml analysis workflow.

### Data importing

In this step, raw phenotypic data is imported into the R workspace, and metadata information is generated (Fig. 1). Information about the field trial, data collection, experimental design, and more are given in section 1 of the sample pre-processing HTML file available on GitHub (https://github.com/whussain2/Analysis-pipeline). The raw phenotypic data imported can also be visualized in the table format in the report. Interestingly, the table generated for raw phenotypic data is highly interactive and can be easily managed, searched, and sorted like a mini excel sheet (section 2 in the sample pre-processing HTML file). Further, the table generated can be easily exported in various formats or printed directly within the document. The raw phenotypic data used for the demo purpose in this study comes from one of the rainfed breeding program trials, which were evaluated in alpha lattice design with two replications and six blocks across multiple environments in Africa with a total of 200 unique genotypes per environment. Besides replications and blocks, row and column information is noted down to account for the spatial trends.

### Data pre-processing and quality check

Data pre-processing involving the quality control of data is the most critical and complex step in data analysis. The workflow to pre-process and check the quality of data is given on a pre-processing HTML file available on GitHub page (https://github.com/whussain2/Analysis-pipeline). We provide a series of quality control steps (Fig. 1) to ensure better data quality and advance quality phenotypes for downstream analysis to get more reliable and accurate estimates or predictors. In general, data pre-processing steps include checking for noise, i.e., removing outliers, errors, or missing data, removing corrupt or inaccurate records, checking for normality assumptions for more reliable estimates, and looking for linearity or co-linearity for best model fit. Briefly, the steps mentioned in the document are:

- *Missing data*: Here, the raw phenotypic data is visualized, and the proportion of missing data is visualized (section 3.1 in the sample pre-processing HTML file). Data can be filtered based on a certain proportion of missing data. For example, we dropped the trials having more than 20% of the data in this demonstration.
- *Descriptive statistics*: In this step, phenotypic data points are summarized as mean, mode, coefficient of variation, the standard deviation for a given variable. Descriptive statistics helps to understand data much better and is the initial step to draw conclusions from the data and plan the model fitting. In addition, the descriptive statistics components may give a clue about the possible errors in the data. For example, the coefficient of variation (CV) calculated in this section can be used to measure variability for a given trait, determine the best plot size in uniformity trials, measure the stability of phenotypes, or measure variation in other individuals or populations attributes [7].
- *Generate heatmaps of field*: In this step, interactive heatmaps of experimental field design are plotted to check for the field's spatial trends and the trend in the missing data. The presence of spatial effects in the data means that advanced models accounting for the spatial effects is required to get better estimates or predictions (section 3.3 in the sample pre-processing HTML file)
- *Data visualization*: In this step, data is visualized using box plots, histograms, and QQ plots. Histograms show the data distribution and ideas about normality assumptions, and QQ plots depict quantiles of the datasets and assess correlated errors among data points for a given variable (section 3.4 in the sample pre-processing HTML file). Similarly, box plots shown in the data are an excellent technique to visualize the data distribution, dispersion, outlier detection, and trait variation. We also interactively presented the boxplots so that more information is obtained that is hidden in static boxplots.
- *Filter for outliers*: In this step, outliers are identified and filtered using the Bonferroni-Holm test [8, 9]. Bonferroni-Holm test is more powerful and reliable when dealing with either small or large data sets. It can identify outliers based on the significance of residuals (section 3.5 in a sample pre-processing HTML file).
- *Reliability of trial*: Based on yield data, we also look at the reliability of each trial or environment as a quality criterion. Any experimental trials having reliability lower than 0.2 are dropped from the analysis (section 3.6 in the sample pre-processing HTML file). More details on reliability and how to calculate it are given in the sample pre-processing HTML file.

### Data analysis

The data analysis demonstrated here is divided into single or separate-trial analysis and multi-environment trial analysis. We demonstrated data analysis of MET both in the ASReml-R package [10] and lme4 R package [11]. We also demonstrated the analysis using marker data and extracting the genomic estimated breeding values (GEBVs) using the gBLUP model.

#### Data analysis in ASReml-R package

- Single-trial analysis

In single-trial analysis, each trial or environment is analyzed separately. Data for a given variable is analyzed using a mixed model approach in the ASReml-R package. Data analysis includes basic mixed models accounting only for experimental design factors (blocks and replications here) and advanced mixed models accounting for experimental design factors and spatial effects or trends [12–14]. In total, five mixed models were implemented to analyze the data and correct for the experimental design factors and spatial trends (correlated residuals across the field dimensions). More advanced models can be used in phenotypic data analysis to account for the spatial trends [13, 15]. However, in this demo, we just showed examples of five mixed models. The best model selected based on AIC values (lower the AIC value better the model) and residual plot information [16] was used to extract the Best Linear Unbiased Predictors (BLUPs). In the analysis, we used genotypes as a random effect to extract the BLUPs, which are good for the phenotypic selection and ranking of the lines in the breeding programs [17, 18]. However, suppose we are going to use genomic selection or predictions in the breeding program. In that case, it is better to use the genotypes as a fixed effect and extract the BLUEs, which can be used as a response variable in the genomic prediction model to extract the BLUPs or breeding values. The reason to use lines as fixed effects is to avoid double shrinkage if we use genotypes as random effects in both cases [17]. We also demonstrated how to use marker data and genomic relationship matrix to model the phenotypic data and extract the genomic estimated breeding values for each line using single-step genomic selection approach [19, 20].

The details of the five models used in the demonstration are given below and in the sample document of ASReml-R workflow HTML file (section 1.1) available on GitHub.

**Model 1:** In this model, we account for just experimental design factors, blocks and replications and no spatial trends, i.e., correlated residuals across the trial's dimensions (rows and columns). Here in this model, blocks and genotypes are used as random effects. The description of model 1 is as:

$${y}_{ijk}=\mu +{g}_{i}+{r}_{j}+{b}_{jk}+{\epsilon }_{ijk}$$

where, ${y}_{ijk}$ is the effect of *i-th* genotype in *j-*th replication and *k-*th block nested within *j-*th replication; $\mu$ is the overall mean; ${g}_{i}$ is the random effect of the *i-*th genotype; ${r}_{j}$ is the fixed effect of *j-*th replication; ${b}_{jk}$ is the random effect of *k-*th block nested within *j-*th replication; ${\epsilon }_{ijk}$ is the residual error.

Here we assume residuals are independent and identically distributed as $\epsilon \sim iidN(0,{\sigma }_{\epsilon }^{2})$.

**Model 2:** In this model, we account for experimental design factor blocks, replications, rows and columns, and no spatial trends. Blocks, rows, and columns, and genotypes were used as random effects. The description of model 2 is as:

$${y}_{ijklm}=\mu +{g}_{i}+{r}_{j}+{b}_{jk}+{c}_{l}+{ro}_{m}+{\epsilon }_{ijklm}$$

where, ${y}_{ijklm}$ is the effect of *i-*th genotype in the *j-*th replication, *k-*th block nested within *j-*th replication, *l-*th column and *m-*th row; $\mu$ is the overall mean; ${g}_{i}$ is the random effect of the *i-*th genotype; ${r}_{j}$ is the fixed effect of *j-*th replication; ${b}_{jk}$ is the random effect of *k-*th block nested within *j-*th replication; ${c}_{l}$ is the random effect of the *l-*th column; ${ro}_{m}$ is the random effect of the *m-*th row; ${\epsilon }_{ijklm}$ is the residual error.

Here we assume residuals are independent and identically distributed as $\epsilon \sim iidN(0,{\sigma }_{\epsilon }^{2})$.

**Model 3:** In this model, we account for experimental design factors, replications and blocks, and spatial trends, i.e., correlated residuals across rows and columns. Blocks and genotypes were used as random effects. The description of model 3 is as:

$${y}_{ijk}=\mu +{g}_{i}+{r}_{j}+{b}_{jk}+{\epsilon }_{ijk}$$

where, ${y}_{ijk}$ is the effect of *i-*th genotype in *j-*th replication and *k-*th block within *j-*th replication; $\mu$ is the overall mean; ${g}_{i}$ is the random effect of the *i-*th genotype; ${r}_{j}$ is the fixed effect of *j-*th replication; ${b}_{jk}$ is the random effect of *k-*th block nested within *j-*th replication; ${\epsilon }_{ijk}$ is the residual error.

Here, we assume $\epsilon$ is a random effect representing correlated residuals based on the distance between plots along the rows and columns, where $\epsilon \sim N(0,\mathbf{R})$ and **R** is the covariance matrix of $\epsilon$. The difference between this model and model 1 and model 2 described above is the structure of the covariance residuals **R** $={{\sigma }_{\epsilon }^{2}{\varvec{\Sigma}}}_{{\varvec{c}}}\left({\rho }_{c}\right)\otimes {{\varvec{\Sigma}}}_{\mathbf{r}}\left({\rho }_{r}\right)$*.* ${\sigma }_{\epsilon }^{2}$ is the variance of spatially dependent residual, $\left({\rho }_{c}\right)and\ {{\varvec{\Sigma}}}_{\mathbf{r}}\left({\rho }_{r}\right)$ represents the first-order autoregressive correlation matrices and ${\rho }_{c}$ and ${\rho }_{r}$ are the autocorrelation parameters for the columns and rows; $\otimes$ represents the Kronecker product between separable auto-regressive processes of the first order in the row-column dimensions [21–24].

**Model 4**: In this model, we account for experimental design factors, replications and blocks, and spatial trends, i.e., correlated residuals across rows only.

$${y}_{ijk}=\mu +{g}_{i}+{r}_{j}+{b}_{jk}+{\epsilon }_{ijk}$$

where, ${y}_{ijk}$ is the effect of *i-*th genotype in *j-*th replication and *k-*th block within *j-*th replication; $\mu$ is the overall mean; ${g}_{i}$ is the random effect of the *i-*th genotype; ${r}_{j}$ is the fixed effect of *j-*th replication; ${b}_{jk}$ is the random effect of *k-*th block nested within *j-*th replication; ${\epsilon }_{ijk}$ is the residual error.

Here, we assume $\epsilon$ is a random effect representing correlated residual based on the distance between plots along the rows only, where $\epsilon \sim N(0,\mathbf{R})$ and **R** is the covariance matrix of $\epsilon$. Here, $\mathbf{R}={\mathbf{I}}_{c}$. ${\sigma }_{\epsilon }^{2}\otimes {{\varvec{\Sigma}}}_{\mathbf{r}\mathbf{o}}\left({\rho }_{ro}\right)$. ${\sigma }_{\epsilon }^{2}$ is the variance of spatially dependent residual; ${{\varvec{\Sigma}}}_{\mathbf{r}\mathbf{o}}\left({\rho }_{r}\right)$ represents the first-order auto-regressive correlation matrices and ${\rho }_{ro}$ is the auto-correlation parameters for the rows; $\otimes$ represents the Kronecker product between separable auto-regressive processes of the first order in the row dimensions. ${\mathbf{I}}_{\mathrm{c}}$ represents independently and identically distributed variance structure for columns.

**Model 5**: In this model, we account for experimental design factors replications and blocks, and spatial trends i.e., correlated residuals across columns only.

$${y}_{ijk}=\mu +{g}_{i}+{r}_{j}+{b}_{jk}+{\epsilon }_{ijk}$$

where, ${y}_{ijk}$ is the effect of *i-*th genotype in *j-*th replication and *k-*th block within *j-*th replication; $\mu$ is the overall mean; ${g}_{i}$ is the random effect of the *i-*th genotype; ${r}_{j}$ is the fixed effect of *j-*th replication; ${b}_{jk}$ is the fixed effect of *k-*th block within *j-*th replication; ${\epsilon }_{ijk}$ is the residual error.

Here, we assume $\epsilon$ is a random effect that represents correlated residual across columns only, where, $\epsilon \sim N(0,\mathbf{R})$ and **R** is the covariance matrix of$\epsilon$, and $\mathbf{R}={{\sigma }_{\epsilon }^{2}{\varvec{\Sigma}}}_{\mathbf{c}}\left({\rho }_{c}\right)\otimes {\mathbf{I}}_{r}$. ${\sigma }_{\epsilon }^{2}$ is the variance of spatially dependent residual; ${\Sigma }_{\mathrm{c}}\left({\uprho }_{\mathrm{c}}\right)$ represents the first-order autoregressive correlation matrices and ${\rho }_{c}$ the autocorrelation parameters for the columns only; ${\mathbf{I}}_{\mathrm{r}}$ represents independently and identically distributed variance structure for rows.

b)
Multi-environment trial (MET) analysis
  Depending upon the number of environments, MET analysis can be performed using single-stage or stage-wise approaches for analysis [12, 22, 25, 26]. The single-stage analysis is the golden standard to analyze the MET data. However, stage-wise analysis is more appropriate in the experiments or trials with unbalanced data sets, different experimental design factors across trials, and to avoid computational challenges of analyzing a huge number of trials. In a stage-wise or two-step approach, adjusted means are estimated per trial or environment, and weighted adjusted means (associated variance–covariance matrix) are fitted in the second step to get the predicted means for each genotype. This demonstration showed how to perform MET analysis using both the single-stage and two-stage or step analysis. The details on the MET analysis using the ASReml-R package is given on sample ASReml-R workflow HTML file (section 1.2) available on GitHub (https://github.com/whussain2/Analysis-pipeline).
  i) Single-stage approach
    In single-stage analysis, all the trials are analyzed jointly. Here, a joint analysis of MET is performed using a linear mixed model (LMM). The mixed model used is defined as:
    $${y}_{ijkl}=\mu +{g}_{i}+{e}_{j}+ {(ge)}_{ij}+{{r}_{jk}{+b}_{jkl}+\epsilon }_{ijkl}$$
    where, ${y}_{ijkl}$ is the effect of *i-*th genotype is *j-*th environment, *k-*th replication nested within *j-*th environment and *l-*th block nested within *k-*th replication and *j-*th environment; $\mu$ is overall mean; ${g}_{i}$ is the random effect of *i-*th genotype; ${e}_{j}$ is the random effect of *j-*th environment; ${ge}_{ij}$ is the interaction effect of *i-*th genotype with *j-*th environment; ${r}_{jk}$ is the fixed effect of *k-*th replication nested within *j-*th environment, ${b}_{jkl}$ is the random effect of *l-*th block nested within *k-*th replication and *j-*th environment, ${\epsilon }_{ijkl}$ is the residual.
    In the matrix notation the mixed model can be represented as:
    $$\mathbf{y}=\mathbf{X}{\varvec{\upbeta}}+{\mathbf{Z}}_{\mathbf{g}}{\mathbf{u}}_{1}+{\mathbf{Z}}_{\mathbf{b}}{\mathbf{u}}_{2}+{\varvec{\upepsilon}}$$
    where, $\mathbf{y}$ is a vector of phenotypic trait values in all the genotypes; $\mathbf{X}$ is the design matrix of fixed effects of replications; ${\mathbf{Z}}_{\mathbf{g}}$ is the design matrix of genotypes within environments that combine the main effects of genotypes, environments and genotype by environment interactions; ${\mathbf{Z}}_{\mathbf{b}}$ is the random effect of blocks nested within the replications. ${\varvec{\upbeta}}$ is the vector of fixed effects estimates; ${\mathbf{u}}_{1}$, ${\mathbf{u}}_{2}$, ${\varvec{\upepsilon}}$ are the vector of random effects of genotypes, blocks nested within replications, and residuals within environments, respectively.
    Random effects are assumed to be random and normally distributed with zero mean vectors and variance–covariance matrices **B**, **G**, **R**, respectively, such that the joint distribution of these three terms is given as:
    $$\left[\begin{array}{c}{\mathbf{u}}_{1}\\ {\mathbf{u}}_{2}\\ {\varvec{\upepsilon}}\end{array}\right]\sim \left(\left[\begin{array}{c}\mathbf{0}\\ \mathbf{0}\\ \mathbf{0}\end{array}\right],\left[\begin{array}{ccc}\mathbf{G}& \mathbf{0}& \mathbf{0}\\ \mathbf{0}& \mathbf{B}& \mathbf{0}\\ \mathbf{0}& \mathbf{0}& \mathbf{R}\end{array}\right]\right)$$
    **G** is a variance–covariance (VCOV) matrix for the effect of genotypes within environments. For the **G** matrix, different VCOV structures were tested, such as compound symmetry (CS), diagonal (Diag), common genetic correlations (corgh), and FA of order *k*, in which *k* is the number of multiplicative components (FAk). For the **R** matrix, identity and diagonal and spatial trends VCOV structures were tested [27–33].
    Here in this demo, we applied 10 models depending upon the variance–covariance structure of random and residual effects. The brief description of these models is given below:
    **Model 1 and model 2**: Models 1 and 2 were basic models in which we assume the variance for residuals and random effects are independent and normally distributed, and implying genotypes have the same variance over the environments. Genotypic variance and covariance between pairs of environments are homogeneous, which corresponds to compound symmetry (CS) variance–covariance structure in the mixed model.
    **Model 3:** In this model we assume different variances across environments, i.e., heterogeneous error variances across environments.
    **Model 4:** In this model, we assume different variances across environments with spatial variance structure same for all the environments. It is assumed that each environment comprises of a rectangular array of rows(r) and columns (c) with **R** $={{\sigma }_{\epsilon }^{2}{\varvec{\Sigma}}}_{\mathbf{c}}\left({\rho }_{c}\right)\otimes {{\varvec{\Sigma}}}_{\mathbf{r}}\left({\rho }_{r}\right)$. ${\sigma }_{\epsilon }^{2}$ is the variance of spatially dependent residual; ${{\varvec{\Sigma}}}_{\mathbf{c}}\left({\rho }_{c}\right) \ and \ {{\varvec{\Sigma}}}_{\mathbf{r}}\left({\rho }_{r}\right)$ represents the first-order autoregressive correlation matrices and ${\rho }_{c}$ and ${\rho }_{r}$ are the autocorrelation parameters for the columns and rows; $\otimes$ represents the Kronecker product between separable auto-regressive processes of the first order in the row-column dimension.
    **Model 5**: This model assumes different variances across environments with spatial variation structure specific to each environment. The best spatial model defined for each environment structure was obtained from the separate analysis done for each environment, as shown in the above section.
    **Model 6**: In this model, we assume a uniform correlation and heterogeneous genetic variance. Each environment has a unique genetic variance, but there were no correlations between environments.
    **Model 7**: In this model, we assume unique genetic variance in each environment with uniform correlations between environments.
    **Models 8, 9, and 10**: Here, we applied factor analytical models FA of order *k*, in which *k* is the number of multiplicative components ${(FA}_{k})$. In factor analytical model 10, we also assume the spatial variance structures are the same across each environment. More details on factor analytical models can be found in these papers [30–32, 34–38].
  ii) Two-stage approach
    Here in this section, the joint analysis of MET data was performed in two-stages. In the first-stage adjusted means as BLUEs and residuals in each environment were obtained by considering the genotypes as fixed effect. At this step, the adjusted means of genotypes were corrected for the experimental design factors, including blocks and replications and spatial trends in each environment. The model used follows as:
    $${y}_{ijk}=\mu +{\mathrm{g}}_{i}+{r}_{j}+{{ b}_{jk}+\epsilon }_{ijk}$$
    where, ${y}_{ijk}$ represents adjusted means for *i-*th observation in *j-*th replication and *k-*th block nested within *j-*th replication; μ is the overall mean; ${g}_{i}$ is the effect of *i-*th genotype; ${r}_{j}$ is the effect of *j*-th replications; ${b}_{jk}$ is the effect of *k-*th block nested within *j-*th replication; ${\epsilon }_{ijk}$ is the residual error.
    Here, we assume $\epsilon \sim N(0,\mathbf{R})$ and **R** is the covariance matrix of $\epsilon$ and **R** $={{\sigma }_{\epsilon }^{2}{\varvec{\Sigma}}}_{\mathbf{c}}\left({\rho }_{c}\right)\otimes {{\varvec{\Sigma}}}_{\mathbf{r}}\left({\rho }_{r}\right)$. ${\sigma }_{\epsilon }^{2}$ is the variance of spatially dependent residual; ${{\varvec{\Sigma}}}_{\mathbf{c}}\left({\rho }_{c}\right)\ and \ {{\varvec{\Sigma}}}_{\mathbf{r}}\left({\rho }_{r}\right)$ represents the first-order autoregressive correlation matrices and ${\rho }_{c}$ and ${\rho }_{r}$ are the autocorrelation parameters for the columns and rows; $\otimes$ represents the Kronecker product between separable auto-regressive processes of the first order in the row-column dimensions.
    In the second-stage, a mixed model was fitted across each environment using the BLUEs obtained from the first-stage as response variable. The model used follows as:
    $${y}_{ij}=\mu + {g}_{i}+{e}_{j}+ {(ge)}_{ij}+ {\epsilon }_{ij}$$
    where, ${y}_{ij}$ is the BLUE value for *i-*th observation in *j-*th environment; μ is the overall mean; ${g}_{i}$ is the random effect of *i-*th genotype; ${e}_{j}$ is the random effect of *j-*th environment; ${ge}_{ij}$ is the interaction effect of *i-*th genotype with *j-*th environment; ${\epsilon }_{ij}$ is the residual error.
    Here, we assume the error is known from the first stage. To account for the errors in the second stage, reciprocal of squared standard errors (equal to diagonal of variance–covariance matrix) as absolute weights were used, thus constraining the residual variance to one. This procedure of obtaining weights is thoroughly described in Method 2 given in [39].

#### Data analysis in lme4 R package

Phenotypic data modeling is also demonstrated in the lme4 R package, an open-source R package for users who don’t have access to the commercial ASReml-R package. In the lme4 R package, data analysis is again divided into two methods of separate analysis and MET analysis. The details on the analysis in lme4 are available in the lme4 R workflow HTML file available on GitHub (https://github.com/whussain2/Analysis-pipeline). Unfortunately, we cannot reproduce all the analysis depicted in ASReml analysis due to the limitation lme4 has in performing mixed-model analysis.

The description of models used in lme4 for separate and MET analysis is given below:

**Model 1. lme4:** For the separate analysis following mixed model was used. This is equivalent to the basic model 1 used in ASReml analysis. The model followed as:

$${y}_{ijk}=\mu +{g}_{i}+{r}_{j}+{b}_{jk}+{\epsilon }_{ijk}$$

where, ${y}_{ijk}$ is the effect of *i-*th genotype in *j-*th replication and *k-*th block within *j-*th replication; $\mu$ is the overall mean; ${g}_{i}$ is the random effect of the *i-*th genotype; ${r}_{j}$ is the fixed effect of *j-*th replication; ${b}_{jk}$ is the random effect of *k-*th block within *j-*th replication; ${\epsilon }_{ijk}$ is the residual error.

Here, we assume residuals are independent and identically distributed as $\epsilon \sim iidN(0,{\sigma }_{\epsilon }^{2})$

**Model 2. lme4:** For the combined analysis following mixed model was used in lme4:

$${y}_{ijkl}=\mu + {g}_{i}+ {e}_{j}+(g{e)}_{ij}+ {r}_{jk}+{b}_{jkl}+{\epsilon }_{ijkl}$$

where, ${y}_{ijk}$ is the effect of *i-*th genotype in *j-*th environment, k-th replication within *j-*th environment and *l-*th block within *k-*th replication within *j-*th environment; $\mu$ is the overall mean; ${g}_{i}$ is the random effect of the *i-*th genotype; ${e}_{j}$ is the fixed effect of *j-*th environment; $g{e}_{ij}$ is an interaction effect of *i-*th genotype in *j-*th environment; ${r}_{jk}$ is fixed effect of *k-*th replication within genotype *j-*th environment; ${b}_{jkl}$ is the random effect of *l-*th block nested within *k-*th replication and *j-*th environment; ${\epsilon }_{ijkl}$ is the residual error.

The models described above are equivalent to the MET model used in ASReml-R, except we are not modeling any spatial variation here in lme4 as was done in the ASReml-R package.

#### Analysis by incorporating marker data

In the crop breeding programs, it is now a routine to integrate the marker data with phenotypic data to predict the genetic merit of individuals in the framework of mixed-model equations by incorporating a genomic relationship matrix (GRM) constructed by using marker data. This section demonstrated how to extend the phenotypic data analysis to marker-based analysis using a relationship matrix. Here, we show an example of how to fit the gBLUP model to get the genomic estimated breeding values (GEBV). More details on other predictive-based models using marker data can be found in these articles [17, 40, 41, 41–44]. In gBLUP genomic relationship matrix (GRM) based on marker, data is used, and GRM defines similarity or the covariance between genotypes or individuals at the genomic level. More details on how to fit the gBLUP model are given in the ASReml-R workflow HTML file (section 1.3). Briefly, here we are providing general model details and how to construct GRM.

In the matrix notation, the gBLUP model is described as:

$${\varvec{y}}=\mathbf{X}{\varvec{\upbeta}}+{\mathbf{Z}}_{\mathbf{g}}{\mathbf{u}}_{\mathbf{g}}+{\mathbf{Z}}_{\mathbf{b}}{\mathbf{u}}_{\mathbf{r}\mathbf{b}}+{\mathbf{Z}}_{\mathbf{e}}{\mathbf{u}}_{\mathbf{e}}+{\varvec{\upvarepsilon}}$$

where, $\mathbf{y}$ is a vector of individual phenotypes; $\mathbf{X}$ is a design matrix of replications; ${\varvec{\upbeta}}$ is a vector of fixed effects of replications; ${\mathbf{Z}}_{\mathbf{g}}$ is a design matrix of marker effects; ${\mathbf{u}}_{\mathbf{g}}$ is a vector of random marker effects; ${\mathbf{Z}}_{\mathbf{r}\mathbf{b}}$ is a design matrix of non-genetic block effects nested within replications; ${\mathbf{u}}_{\mathbf{r}\mathbf{b}}$ is a vector of random block effects; ${\mathbf{Z}}_{\mathbf{e}}$ is a design matrix of non-genetic random effect of environments and genotype x environment interactions; ${\mathbf{u}}_{\mathbf{e}}$ is a vector of main environment and interaction effects; ${\varvec{\upvarepsilon}}$ is the vector of residual errors.

Further, we assume random effects are normally distributed with zero mean vectors and variance–covariance matrices **G**, **B**, **R** as described in single-stage approach of MET analysis above. Here, the expected variance of markers is given as ${\mathrm{Var}(\mathbf{u}}_{\mathbf{g}})$=${\sigma }_{g}^{2}\mathbf{G}$**,** where $\mathbf{G}$ is genomic or kinship covariance matrix of n x m dimensions (n is no. of markers and m is no. of individuals) representing the genomic similarity of individuals.

Genomic (G) matrix or GRM [37, 39] is constructed using the following equation:

$${\varvec{G}}=\frac{\mathbf{X}\acute{{\mathbf{X}}}}{\mathrm{n}}$$

Here **X** is a scaled and centered matrix of marker data, $\acute{{\mathbf{X}}}$ transpose of **X** matrix, and *n* is the number of total markers or columns in marker data.

### Results

This section demonstrated how to extract the results from the separate analysis or MET analysis using either the ASReml-R package or lme4 R packages. Users can extract the specific results depending upon the objectives. In a separate analysis, we showed how to pull BLUPs, variance components, heritability, ANOVA, and variogram to check for spatial trends for the trait using the best model. In MET analysis, besides these results mentioned above, we used the ASExtras4 R package (https://mmade.org/asextras4/) to extract additional results, including correlation and covariance matrix, G x E BLUPs, PCA biplot, and latent regression figures to check the stability of genotypes (**Fig. ****2**). For more details on these results, check the HTML workflows of ASReml and lme4 analysis available on GitHub.

Additionally, we demonstrated how to extract the heritability and generalized heritability [45] using different approaches. For example, we are dealing with spatial or complex models in this data, so calculating heritability based on the method described by [17, 45] is used to estimate heritability. Briefly, for complex residual structures and unbalanced experimental designs, heritability estimation is given by equation ${H}_{c}=1-\frac{{\overline{V} }_{BLUP}}{{2\sigma }_{g}^{2}}$, where ${\overline{V} }_{BLUP}$ is a mean–variance difference of two BLUPs and ${\sigma }_{g}^{2}$ is a variance of genotypes. Note that this definition of heritability is related to the reliability of breeding value predictions.

Further BLUPs extracted here are used to rank the genotypes for making selections in breeding decisions. In lme4 R package analysis, ANOVA, variance components, fixed effect as BLUEs, random effect as BLUPs, and heritability were extracted. More details on this are given in the sample lme4 R workflow HTML document available on GitHub (https://github.com/whussain2/Analysis-pipeline).

## Converting analysis workflow into a document

**Fig. 3:**
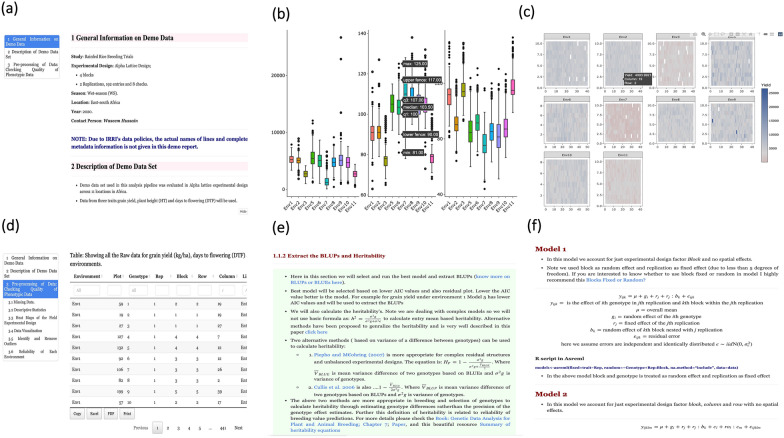
Screenshots of some of the features in the report. **a** General information and table of content for easy navigation of the document. **b**, **c** Interactive heatmap and box plot. **d** Example of interactive tables, which can be managed and exported in various formats. More features and details can be found in the sample HTML files

One of the biggest challenges in data analysis is reporting it and presenting it in a well-documented format for better understanding and making breeding decisions. In data analysis, the ‘copy and paste’ system is mostly used to report the results, which is time-consuming and highly error-prone. Thus, the situation demands a unique technique that converts the analysis workflow explicitly into a report or document for easy interpretations, understanding, and sharing. Here, we not only reported the analysis workflow as described above but also demonstrated how this workflow could be re-designed into a reproducible document for better interpretation, visualization, and seamless sharing with partners. The generated report is the state-of-the-art implementation of an analysis workflow with a description of R scripts and results with interpretations embedded as one unified document. A sample document is available in the GitHub repository at https://github.com/whussain2/Analysis-pipeline. The main features of the document are:

1. The analysis pipeline described and given in Fig. 1 is converted into a highly reproducible document, and the same report and analysis pipeline can be generated anytime when required. The sample source codes of the analytical pipeline and demo data set can be directly downloaded from the GitHub repository (https://github.com/whussain2/Analysis-pipeline). The instructions on how to run the analysis pipeline on a local computer are given on the GitHub repository page.
2. Any new data and editing/corrections to the existing pipeline can be done by simply re-knitting the R markdown ‘*.Rmd*’ document (https://rmarkdown.rstudio.com/articles_intro.html). This analytical pipeline avoids manually updating or generating reports or PowerPoint slides, which are otherwise highly prone to errors and time-consuming.
3. The document includes metadata (information about the field trial design, data collection, experimental design, and more) at the beginning for quick identification, location, and association of data and analysis at any given time (Fig. 3a).
4. The document is well structured and organized. For example, the document is divided into sections with headings and subheadings to increase accessibility and cognition. The table of contents is always visible in the document making it faster and easier to navigate within a document (Fig. 3a). Additionally, readers have the flexibility to hide the sections for better readability and accessibility.
5. The document is currently generated in HTML, which upon download can be easily opened in any browser without requiring any access to the internet. Further, HTML files can be shared easily and hosted on websites for easy sharing and future use.
6. The graphics in the document are highly dynamic and interactive. Simply hovering a cursor on the plot will display the additional hidden information, which is impossible in static pictures. For example, the box plots and heatmaps of experimental field design to visualize spatial trends are highly dynamic and interactive (Fig. 3b and c). Additionally, graphics can be easily exported to the local drive.
7. The output generated in the form of tables is highly dynamic and interactive. The tables generated can be easily managed, searched, and sorted like a mini excel sheet (Fig. 3d). Interestingly, tables can also be exported in various formats or printed directly within the document. The tables and result outputs being in the same file completely avoids the option of saving the files on computers and digging into them to extract useful information in making presentations or in undertaking breeding decisions.
8. Complete description and details of scripts, procedures, and methods used for analysis are elaborated in the same document. Results generated in the document in the form of figures and or tables have been thoroughly described to aid in the interpretation and better understanding. Hyperlinks have been embedded in the required sections to help in understanding the concepts and add knowledge to the users. For example, web sources on how to interpret the box plots; methods used to calculate heritability with complex models; spatial analysis modeling, and much more have been hyperlinked in the document

## Conclusions

Crop breeding trial analysis and procedures are well established in the literature; however, putting them into an end-to-end analysis workflow with detailed descriptions and explanations is not available. A helpful guide and tutorial to thoroughly understand the phenotypic data analysis is a crucial requirement in breeding programs. Here, we took an initiative to modernize the data analysis of IRRI’s RRB program, which can be easily put into practice and will be of great use to the crop breeding communities having full-fledged breeding programs. We believe this will serve a helpful guide specifically for researchers or plant breeders who have little knowledge about phenotypic data analysis. We reported the workflow and analytical pipeline and gave step-by-step instructions and explanations on how to analyze the phenotypic data in the best possible way for making the right breeding decisions. Conclusively, we reported end-to-end implementation of phenotypic data analyses of plant breeding field trials and re-design it into a reproducible document for easy sharing, understanding, and interpretation. In the future, we look forward to incorporating predictive analytics based on higher advanced statistical modeling and big data.

## Data Availability

The datasets and R scripts used to run all the analysis demonstrated in this study are available on the GitHub page at https://github.com/whussain2/Analysis-pipeline.

## Abbreviations

BLUPs: Best linear unbiased predictions
BLUEs: Best linear unbiased estimation
HTML: Hypertext markup language
MET: Multi-environment trial
NARES: National agricultural research and extension systems
